# Supplementary material for: TIR-like NADases act in bacterial immunity and the RNA vault
Source: bioRxiv. 2026 May 5:2026.05.01.722283. Preprint. [Version 1] doi: 10.64898/2026.05.01.722283 (PMC13174449; doi:10.64898/2026.05.01.722283)
Supplement: 2 [file NIHPP2026.05.01.722283v1-supplement-2.pdf]

bioRxiv preprint doi: https://doi.org/10.64898/2026.05.01.722283; this version posted May 5, 2026. The copyright holder for this preprint (which was not certified by peer review) is the author/funder, who has granted bioRxiv a license to display the preprint in perpetuity. It is made available under aCC-BY 4.0 International license.

Supplemental Figures

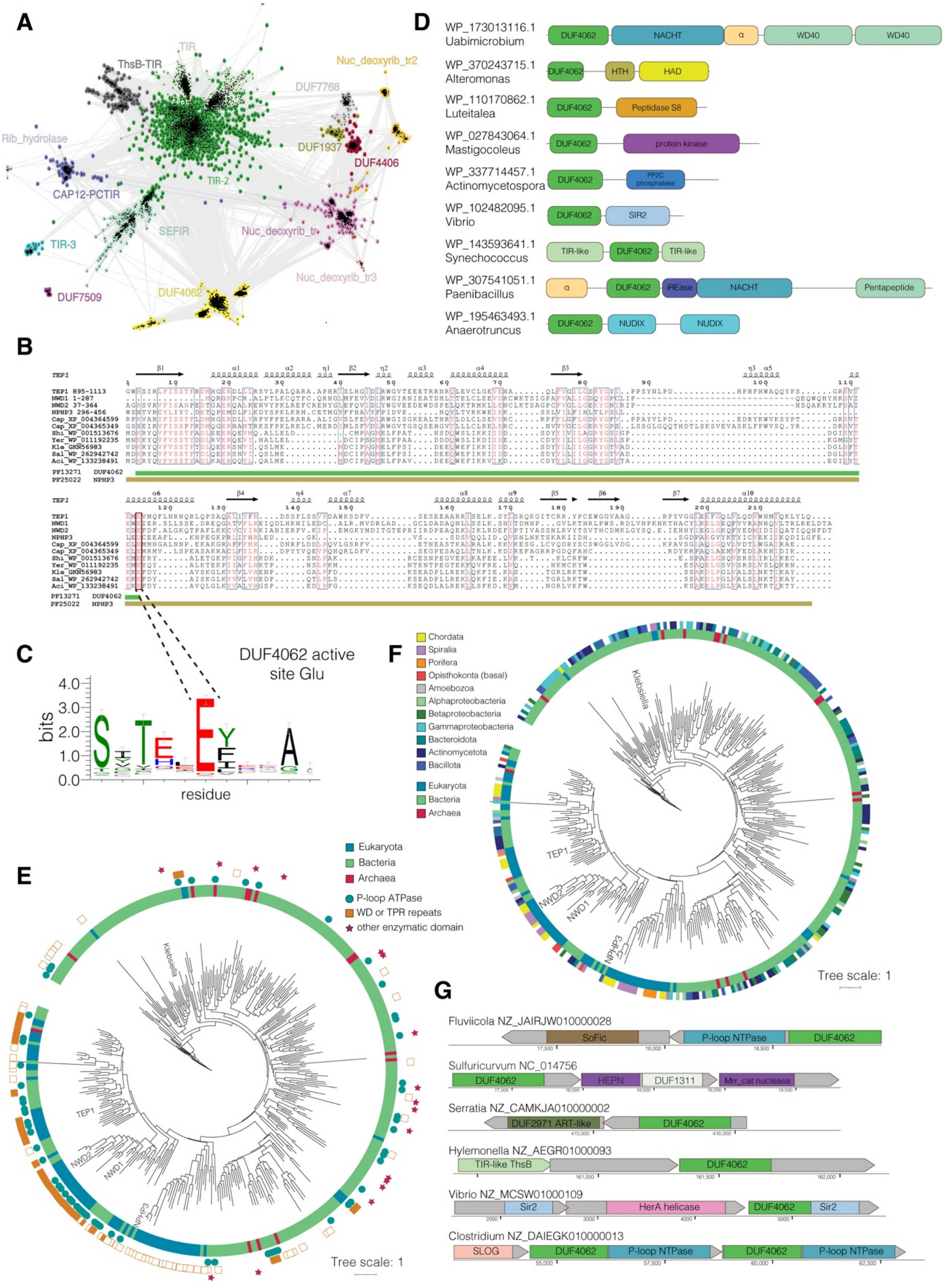

bioRxiv preprint doi: https://doi.org/10.64898/2026.05.01.722283; this version posted May 5, 2026. The copyright holder for this preprint (which was not certified by peer review) is the author/funder, who has granted bioRxiv a license to display the preprint in perpetuity. It is made available under aCC-BY 4.0 International license.

Figure S1. DUF4062 is a TIR-like domain family found in eukaryotes and bacterial anti-phage defense islands.

(A) CLANS sequence similarity network of representative TIR-like and NDT superfamily proteins (Pfam clans CL0173/STIR and CL0498/Nribosyltransf; BLAST E ≤ 0.01). Each point represents a single protein sequence, and distances reflect relative sequence similarity. As in **Figure 1A**, with nodes colored by family.

(B) Multiple sequence alignment of selected DUF4062 domains. Shown are the four human DUF4062 domains, the *Capsaspora* (Cap) DUF4062 domain, and bacterial DUF4062-containing proteins used in the BASEL screen in **Figure 1C**: *Shigella* (Shi), *Yersinia* (Yer), *Klebsiella* (Kle), *Salmonella* (Sal), and *Acinetobacter* (Aci). NCBI sequence identifiers are shown. A schematic of secondary structure based on the human TEP1 AlphaFold model is shown at the top, whereas the extent of domain annotations in the UniProt database, DUF4062/PF13271 in human TEP1 (green) and NPHP3/PF25022 (olive), is shown at the bottom.

(C) Sequence logo depicting the conservation of the DUF4062 active site.

(D) Phylogenetic tree of DUF4062 domains, as in **Figure 1C**, annotated with auxiliary domains present in the full-length proteins.

(E) Domain architectures of selected DUF4062 proteins containing auxiliary enzymatic domains.

(F) Phylogenetic tree of DUF4062 domains, as in **Figure 1C**, annotated with selected eukaryotic and bacterial taxa.

(G) Selected operons comprising DUF4062 proteins and associated proteins containing domains implicated in anti-phage defense.

See also **Figure 1**

bioRxiv preprint doi: https://doi.org/10.64898/2026.05.01.722283; this version posted May 5, 2026. The copyright holder for this preprint (which was not certified by peer review) is the author/funder, who has granted bioRxiv a license to display the preprint in perpetuity. It is made available under aCC-BY 4.0 International license.

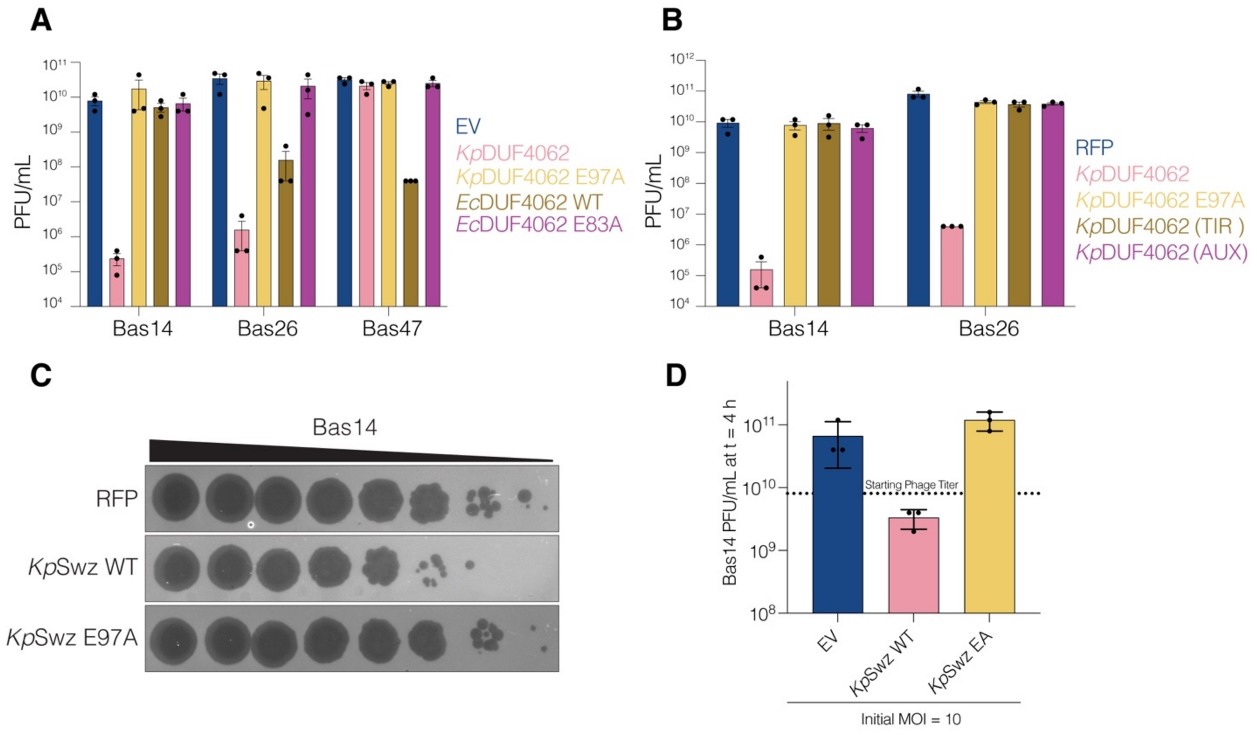

Figure S2. DUF4062 homologs form Swarożyc (Swz), a previously unrecognized antiphage defense system

(A) Quantification of phage plaque assays on BW25113 cells expressing RFP or the indicated *Kp*DUF4062$^{16\text{-}C}$ (*Kp*DUF4062) and *Ec*DUF4062 constructs. Phages were ten-fold serially diluted. Data are presented as mean ± SEM of n = 3 biological replicates.

(B) Quantification of phage plaque assays on BW25113 cells expressing RFP or indicated *Kp*DUF4062 constructs. Phages were ten-fold serially diluted. Data are presented as mean ± SEM of n = 3 biological replicates.

(C, D) Free Bas14 phage collected 200 min post-infection (MOI = 10, **Figure 2G**). Ten-fold serial dilutions of supernatants were plated on BW25113 lawns expressing RFP **(C)** and quantified in **(D)**.

See also **Figure 2**

bioRxiv preprint doi: https://doi.org/10.64898/2026.05.01.722283; this version posted May 5, 2026. The copyright holder for this preprint (which was not certified by peer review) is the author/funder, who has granted bioRxiv a license to display the preprint in perpetuity. It is made available under aCC-BY 4.0 International license.

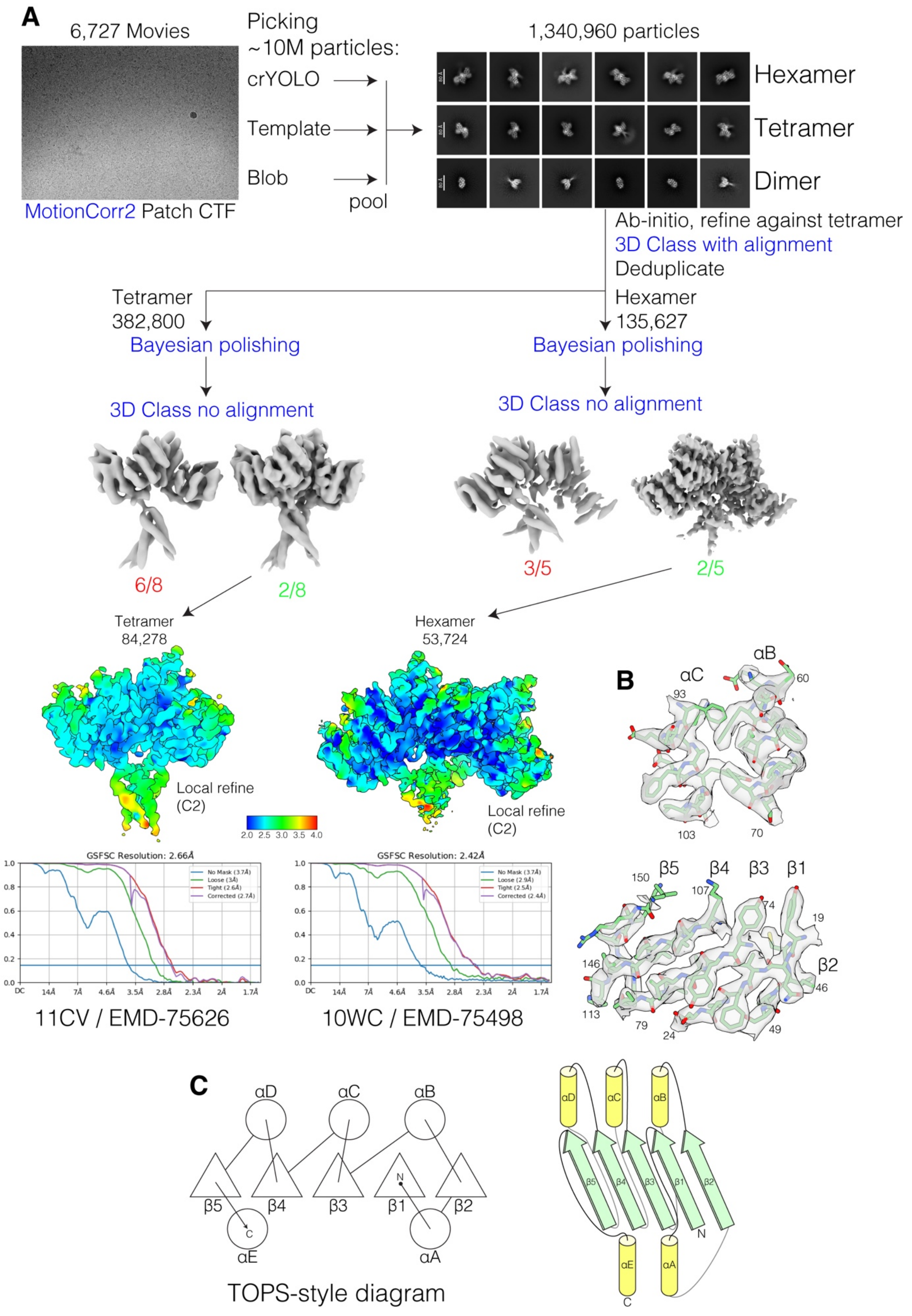

bioRxiv preprint doi: https://doi.org/10.64898/2026.05.01.722283; this version posted May 5, 2026. The copyright holder for this preprint (which was not certified by peer review) is the author/funder, who has granted bioRxiv a license to display the preprint in perpetuity. It is made available under aCC-BY 4.0 International license.

Figure S3. Structural insights into the *Kp*Swz TIR-like domain

(A) Simplified Cryo-EM processing schematic of the KpSwz$^{15\text{-}C,\ E97A}$ dataset.

(B) Density fit of the indicated model portions.

(C) Topology diagrams depicting the secondary structural elements of *Kp*Swz DUF4062 domain.

See also **Figure 3**

bioRxiv preprint doi: https://doi.org/10.64898/2026.05.01.722283; this version posted May 5, 2026. The copyright holder for this preprint (which was not certified by peer review) is the author/funder, who has granted bioRxiv a license to display the preprint in perpetuity. It is made available under aCC-BY 4.0 International license.

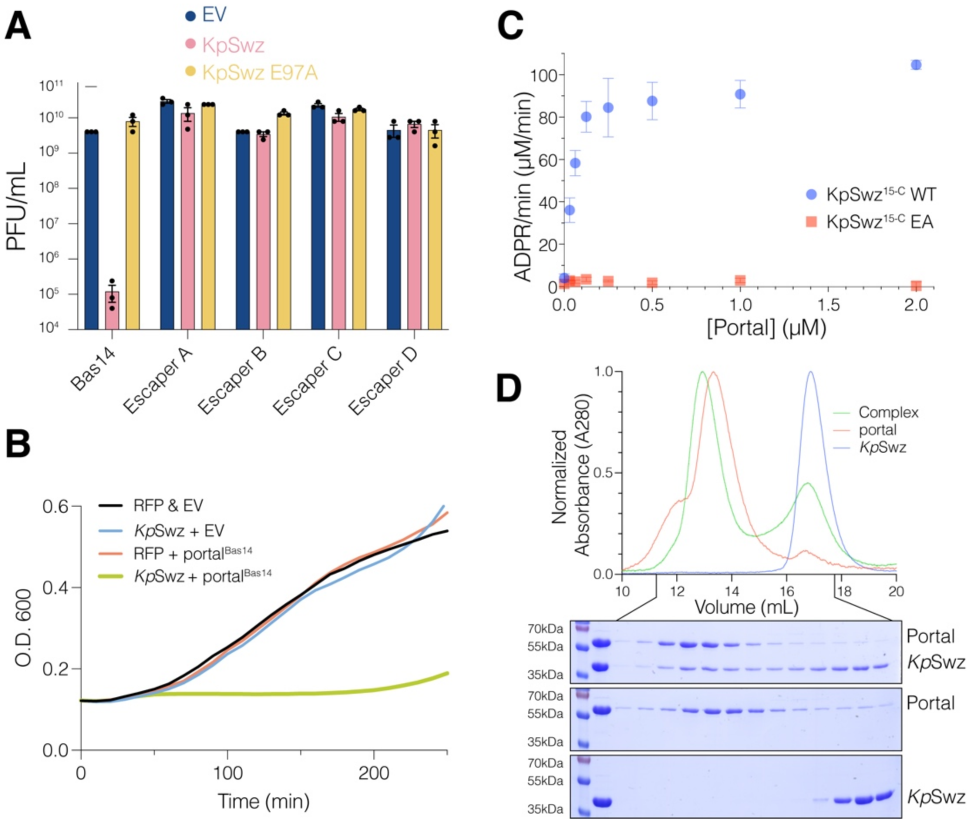

Figure S4. Portal^{Bas14} triggers *Kp*Swz NADase activity and cell death

(A) Quantification of phage plaque assays on BW25113 cells expressing RFP or indicated *Kp*Swz$^{16\text{-}C}$ (*Kp*Swz) constructs. Phages were ten-fold serially diluted. Data are presented as mean ± SEM of n = 3 biological replicates.

(B) Growth of *E. coli* BW25113 cells expressing the indicated *Kp*Swz and portal proteins.

(C) Graph depicting *Kp*Swz$^{15\text{-}C}$ NADase activity in the presence of increasing concentrations of portal^{Bas14}. Reaction products were separated by HPLC-UV$_{260}$ and quantified by the area under the curve using a standard of ADP-ribose. 0.15 μM of KpSwz$^{15\text{-}C}$ was used for 30 minutes at ambient temperature.

(D) FPLC-UV$_{280}$ chromatograms of *Kp*Swz$^{15\text{-}C,\ E97A}$ (blue), Portal^{Bas14} (red), and their complex (green). Proteins were separated on a Superose 6 column, and the indicated fractions were analyzed by SDS-PAGE followed by Coomassie staining. ~150 μg of each protein was used.

See also **Figure 4**

bioRxiv preprint doi: https://doi.org/10.64898/2026.05.01.722283; this version posted May 5, 2026. The copyright holder for this preprint (which was not certified by peer review) is the author/funder, who has granted bioRxiv a license to display the preprint in perpetuity. It is made available under aCC-BY 4.0 International license.

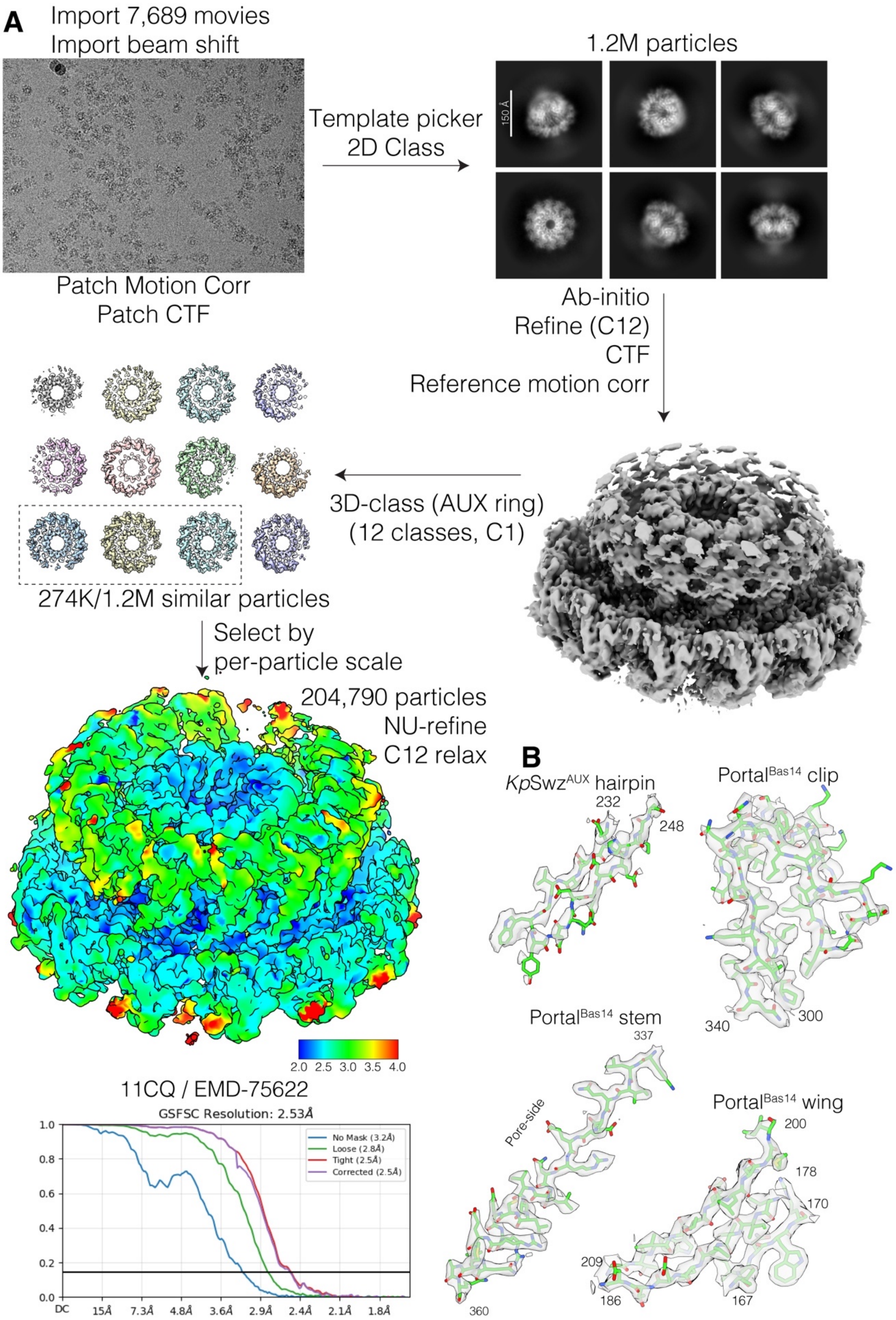

bioRxiv preprint doi: https://doi.org/10.64898/2026.05.01.722283; this version posted May 5, 2026. The copyright holder for this preprint (which was not certified by peer review) is the author/funder, who has granted bioRxiv a license to display the preprint in perpetuity. It is made available under aCC-BY 4.0 International license.

Figure S5: Cryo-EM structure of *Kp*Swz-portal^{Bas14} complex

(A) Simplified Cryo-EM processing schematic of the KpSwz$^{15\text{-}C,\ E97A}$ + portal^{Bas14} dataset.

845 **(B)** Density fit of the indicated model portions.

See also **Figure 4**

bioRxiv preprint doi: https://doi.org/10.64898/2026.05.01.722283; this version posted May 5, 2026. The copyright holder for this preprint (which was not certified by peer review) is the author/funder, who has granted bioRxiv a license to display the preprint in perpetuity. It is made available under aCC-BY 4.0 International license.

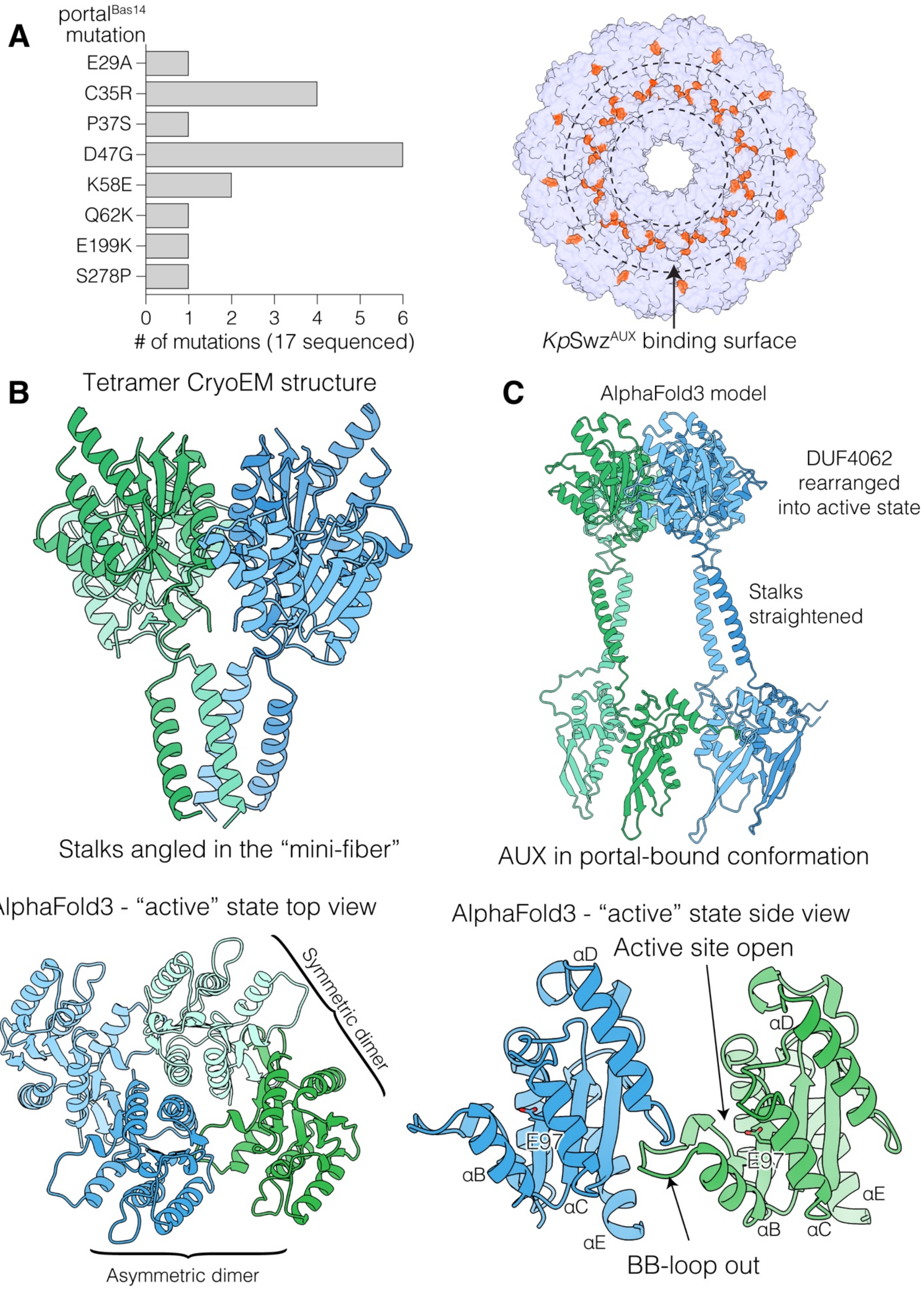

bioRxiv preprint doi: https://doi.org/10.64898/2026.05.01.722283; this version posted May 5, 2026. The copyright holder for this preprint (which was not certified by peer review) is the author/funder, who has granted bioRxiv a license to display the preprint in perpetuity. It is made available under aCC-BY 4.0 International license.

Figure S6. Structural insights into portal^{Bas14} escaper mutations and AlphaFold modeling of the activated *Kp*Swz–portal^{Bas14} complex.

(A) Chart showing the number of mutations identified for the indicated genotype (left) and surface representation of the *Kp*Swz–portal^{Bas14} complex highlighting the positions of the portal^{Bas14} escaper mutations (right).

(B) Cartoon representation of the *Kp*Swz tetramer, depicting the intertwined helical stalk (upper) and a zoomed in view of the active site (lower).

(C) AlphaFold3 modeling of the full-length *Kp*Swz–portal^{Bas14} complex suggests an activation mechanism in which portal^{Bas14} engagement with *Kp*Swz^{AUX} pulls on the helical stalks, aligns them (upper), and ultimately repositions the TIR domains into an active conformation (lower).

See also **Figure 4**

bioRxiv preprint doi: https://doi.org/10.64898/2026.05.01.722283; this version posted May 5, 2026. The copyright holder for this preprint (which was not certified by peer review) is the author/funder, who has granted bioRxiv a license to display the preprint in perpetuity. It is made available under aCC-BY 4.0 International license.

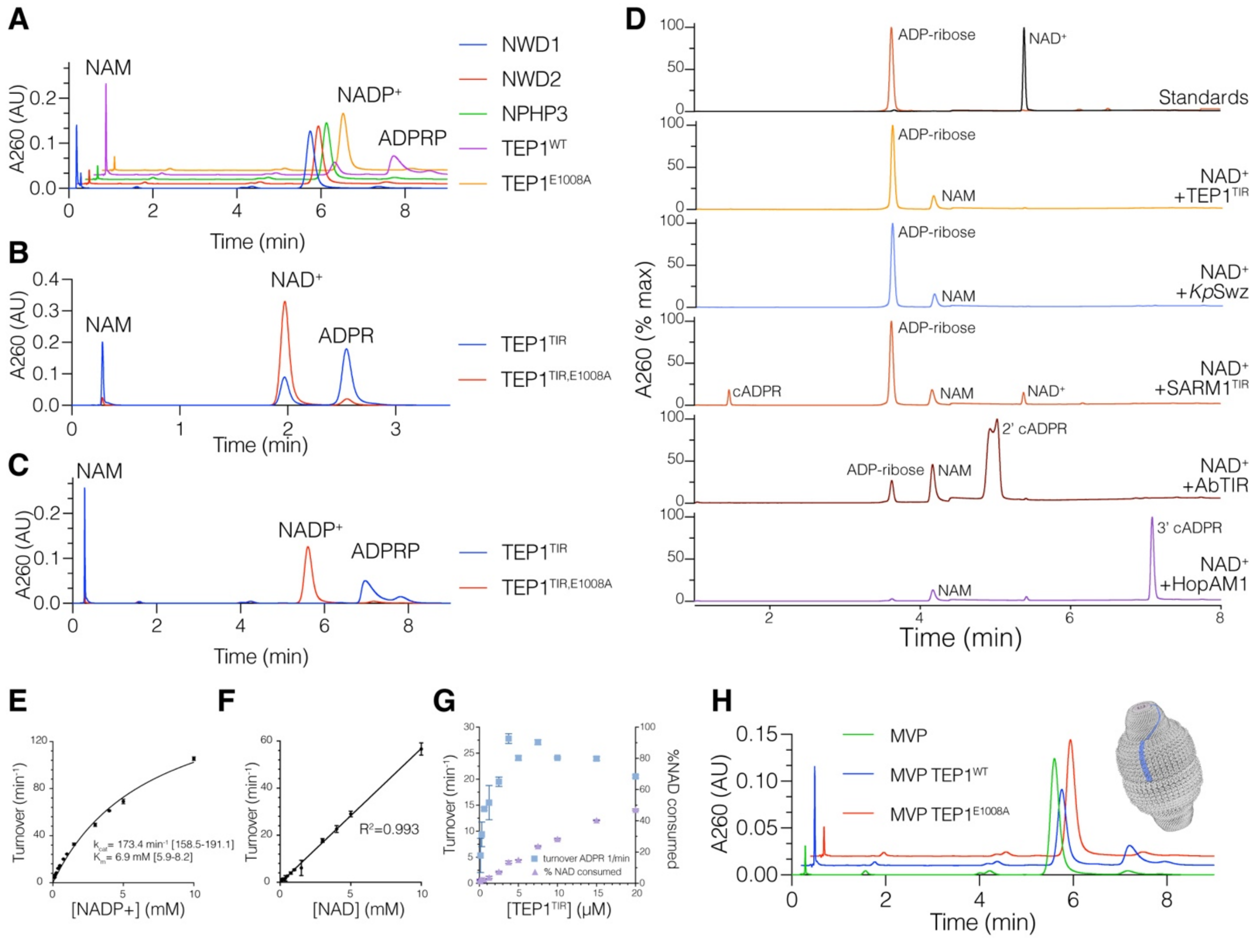

Figure S7. Characterization of TEP1^{TIR} activity.

(A) HPLC traces depicting the reaction products generated after incubation of NADP$^+$ with TEP1, NWD1, NWD2 and NPHP3. Full-length protein preparations were used at 0.5 mg/mL.

(**B, C**) HPLC traces depicting the reaction products generated after incubation of NAD$^+$ **(B)** or NADP$^+$ **(C)** with TEP1^{TIR} or the E1008A mutant.

(**D**) HPLC traces depicting the reaction products generated after incubation of NAD$^+$ for 1 h at 37 °C with 0.3 mg/mL of each TEP1^{TIR}, *Kp*Swz$^{15\text{-}C}$ (*Kp*Swz), SARM1^{TIR}, AbTIR, or HopAM1 (cyclic ADP-ribose; cADPR.

(**E, F**) Kinetic analysis of NADP$^+$ **(E)** or NAD$^+$ **(F)** hydrolysis by 1 μM TEP1^{TIR}, depicting the concentration dependence of NAD(P)$^+$ on reaction rate. The inset shows the

bioRxiv preprint doi: https://doi.org/10.64898/2026.05.01.722283; this version posted May 5, 2026. The copyright holder for this preprint (which was not certified by peer review) is the author/funder, who has granted bioRxiv a license to display the preprint in perpetuity. It is made available under aCC-BY 4.0 International license.

corresponding apparent K_m and k_{cat} values. Reaction products were analyzed and quantified as described in **Figure S4C**.

(G) Graph showing the specific activity of $TEP1^{TIR}$ at increasing concentrations of $TEP1^{TIR}$. Percentage of consumed NAD^+ is plotted on the right. Reaction products were analyzed as in **Figure S4C.** Reactions were conducted for 10 minutes with 10 mM NAD^+.

(H) HPLC traces depicting the reaction products generated after incubation of $NADP^+$ with the RNA vault (0.75 mg/mL) containing either TEP1 (red) or the E1008A mutant (green).

See also **Figure 5**

bioRxiv preprint doi: https://doi.org/10.64898/2026.05.01.722283; this version posted May 5, 2026. The copyright holder for this preprint (which was not certified by peer review) is the author/funder, who has granted bioRxiv a license to display the preprint in perpetuity. It is made available under aCC-BY 4.0 International license.

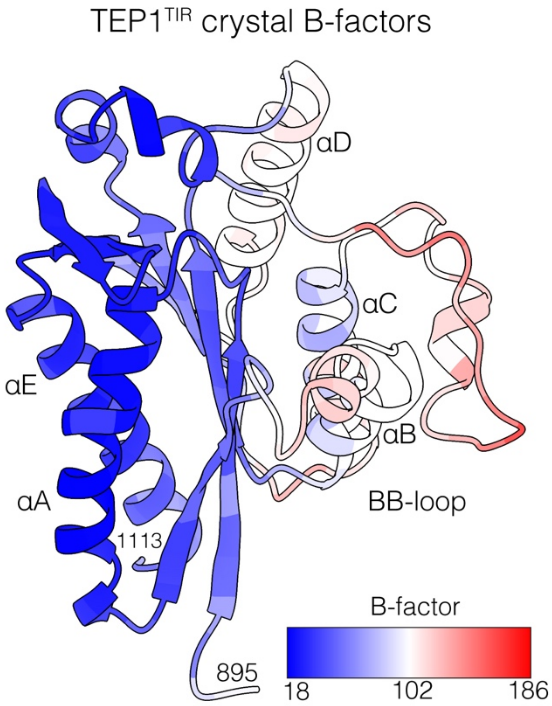

Figure S8. Structure of the TEP1^{TIR} domain.

Crystal structure of the TEP1^{TIR} (895-1113) is shown as a cartoon representation and colored by B-factors at Cα atoms. Note the quality degradation towards the flexible regions of the protein around the active site.

See also **Figure 5**

bioRxiv preprint doi: https://doi.org/10.64898/2026.05.01.722283; this version posted May 5, 2026. The copyright holder for this preprint (which was not certified by peer review) is the author/funder, who has granted bioRxiv a license to display the preprint in perpetuity. It is made available under aCC-BY 4.0 International license.

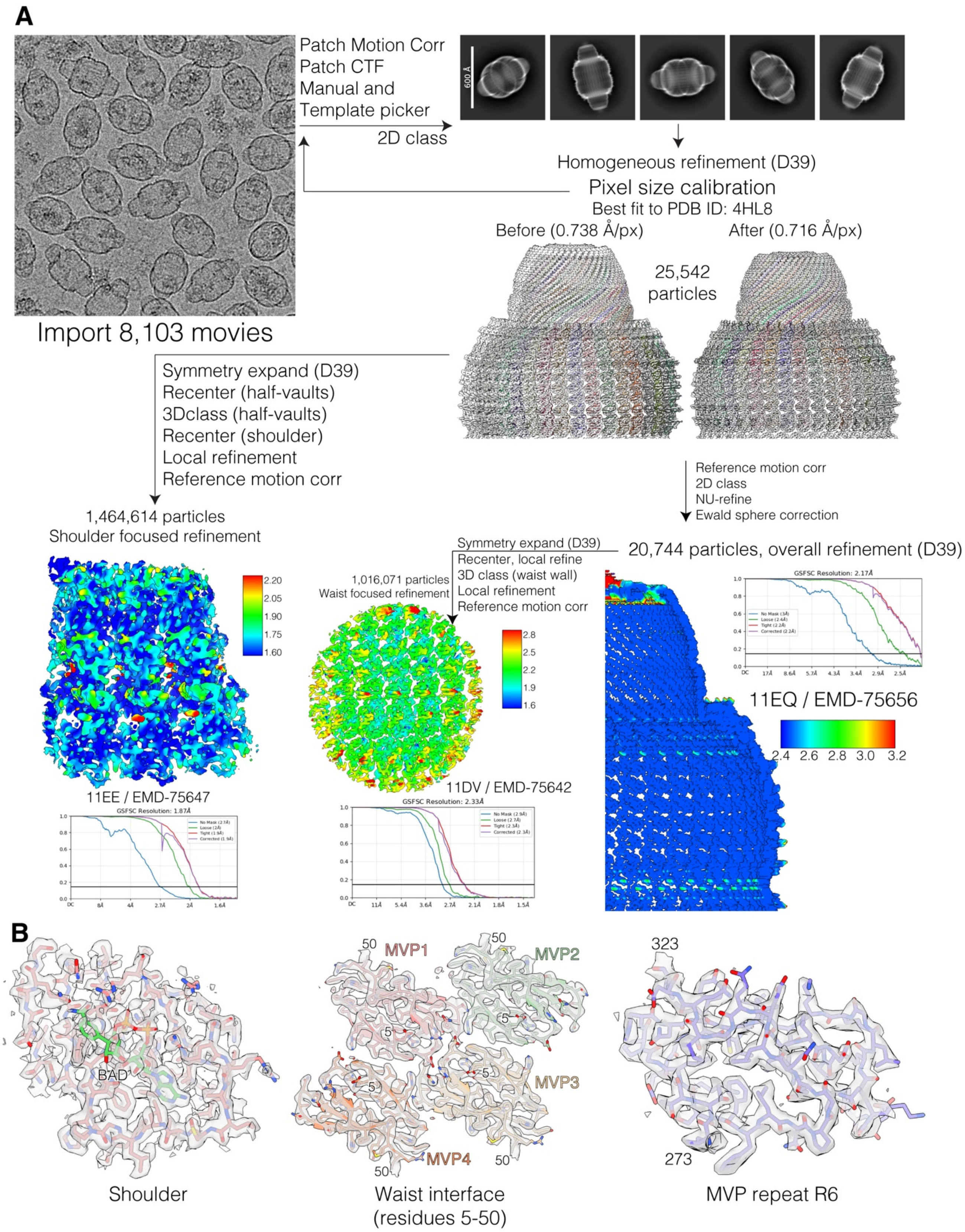

bioRxiv preprint doi: https://doi.org/10.64898/2026.05.01.722283; this version posted May 5, 2026. The copyright holder for this preprint (which was not certified by peer review) is the author/funder, who has granted bioRxiv a license to display the preprint in perpetuity. It is made available under aCC-BY 4.0 International license.

Figure S9. Structure of the RNA vault with TEP1, BAD and AMP-PNP.

(A) Simplified data processing schematic for the dataset.

(B) Example density fit for arbitrarily chosen protein regions.

See also **Figure 6**

bioRxiv preprint doi: https://doi.org/10.64898/2026.05.01.722283; this version posted May 5, 2026. The copyright holder for this preprint (which was not certified by peer review) is the author/funder, who has granted bioRxiv a license to display the preprint in perpetuity. It is made available under aCC-BY 4.0 International license.

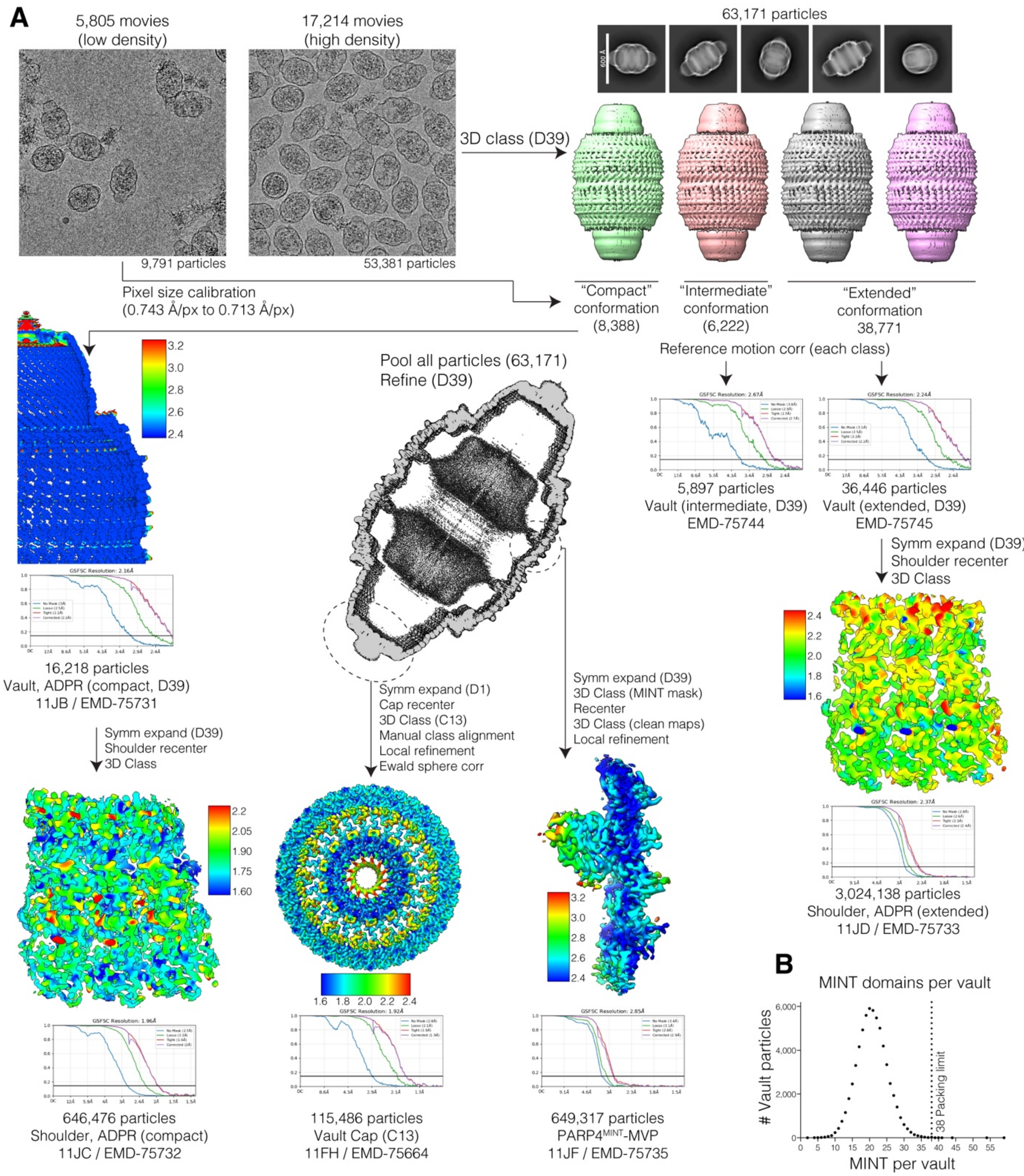

Figure S10. Structure of the RNA vault with PARP4, TEP1 and NADP$^+$.

(A) Simplified data processing schematic for the dataset.

(B) The MINT domains in each vault shell from the initial MINT-focused 3D classification were counted and plotted as a histogram.

See also **Figure 6**

bioRxiv preprint doi: https://doi.org/10.64898/2026.05.01.722283; this version posted May 5, 2026. The copyright holder for this preprint (which was not certified by peer review) is the author/funder, who has granted bioRxiv a license to display the preprint in perpetuity. It is made available under aCC-BY 4.0 International license.

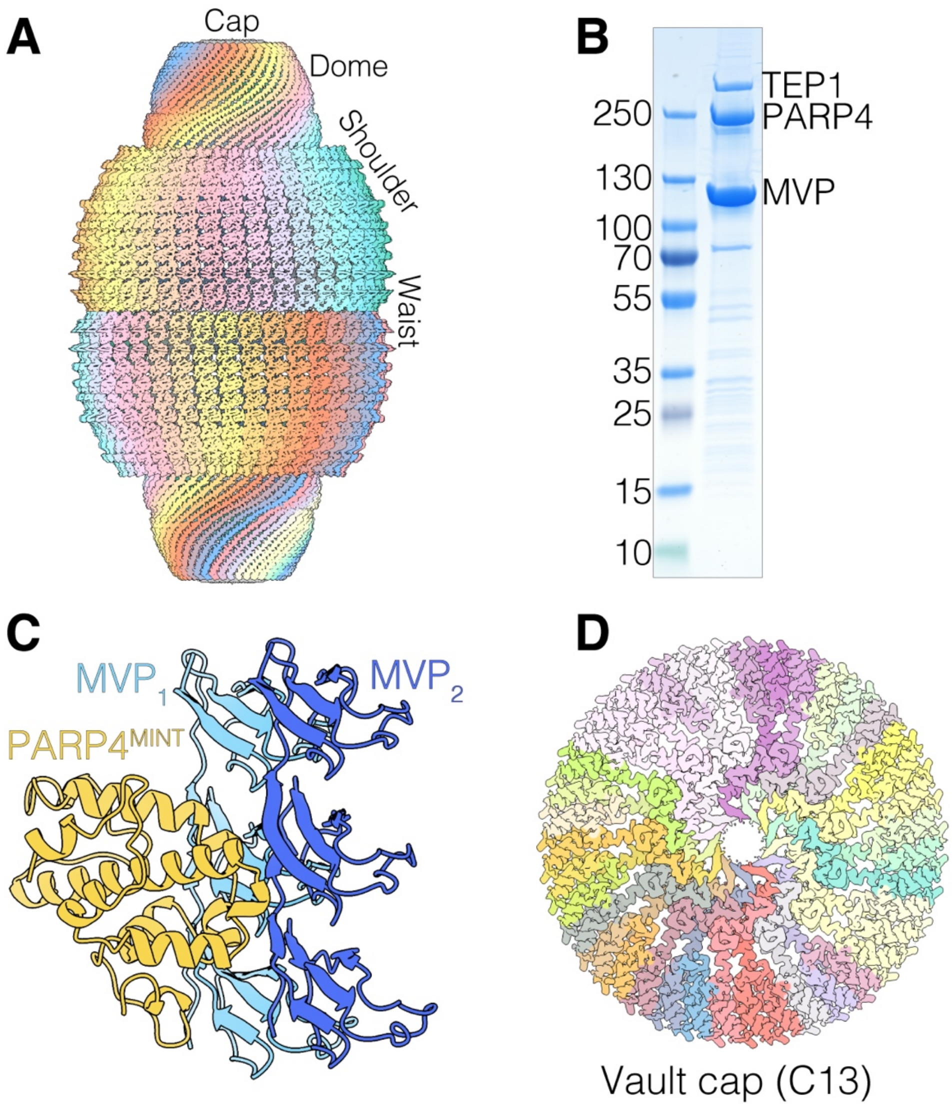

Figure S11. Cryo-EM analysis of reconstituted RNA vaults

(A) Orthographic projection of the human RNA vault shell. The unsharpened Coulombic map of a D39-symmetric vault is shown, colored by MVP monomer.

(B) Coomassie stained SDS-PAGE analysis of the purified reconstituted RNA vault. Bands corresponding to MVP, TEP1, and PARP4 are indicated.

(C) Cartoon depiction of the $PARP4^{MINT}$ domain (yellow) bound between two MVP monomers (blue/light blue).

(D) Coulombic map of the human RNA vault cap, refined with C13 symmetry.

See also **Figure 6**

bioRxiv preprint doi: https://doi.org/10.64898/2026.05.01.722283; this version posted May 5, 2026. The copyright holder for this preprint (which was not certified by peer review) is the author/funder, who has granted bioRxiv a license to display the preprint in perpetuity. It is made available under aCC-BY 4.0 International license.

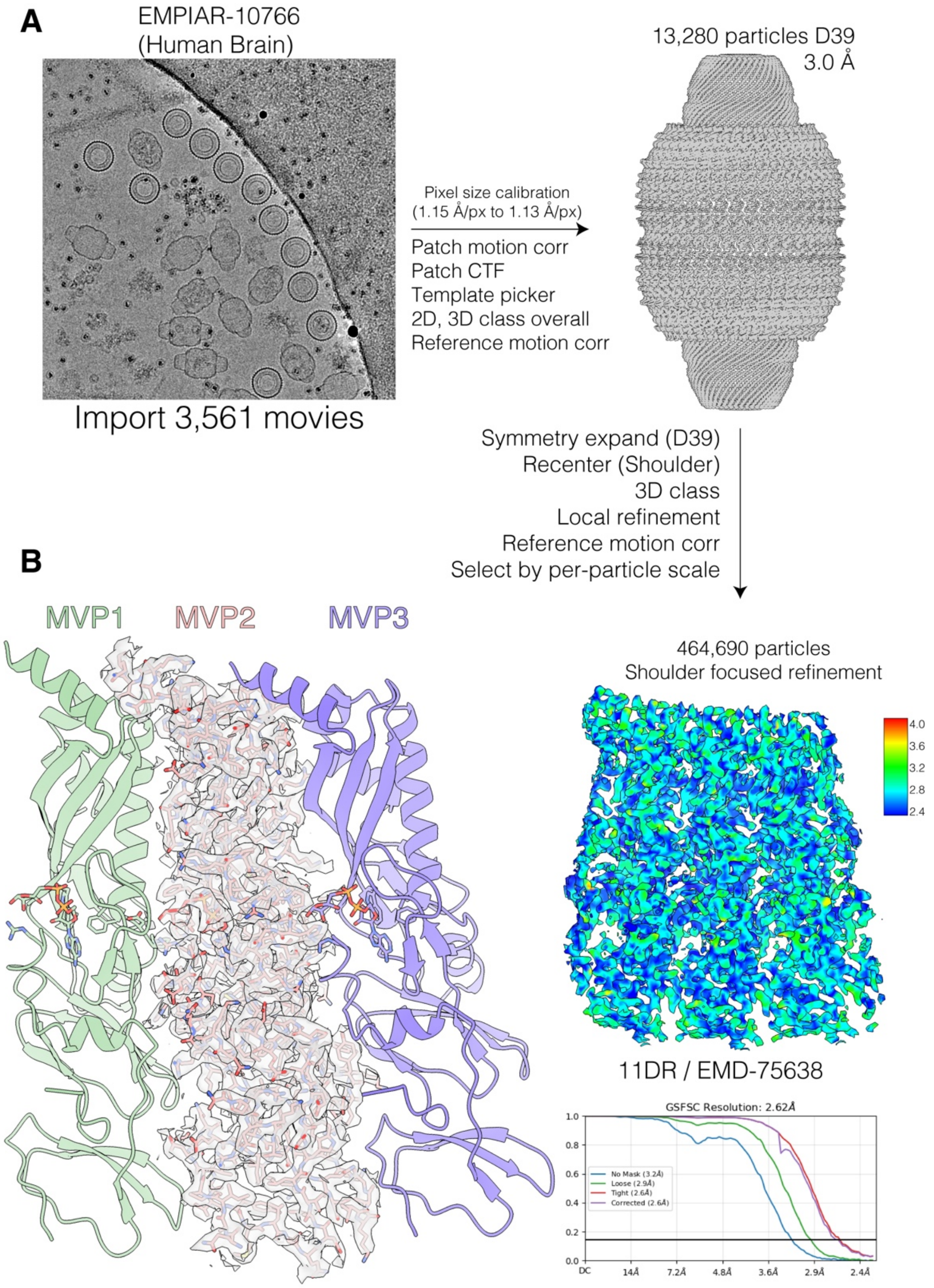

Figure S12. Reprocessing of vaults from human brain reveals a bound ADP-ribose.

(A) Simplified Cryo-EM processing schematic of the EMPIAR-10766 dataset.

(B) Density fit of the model.

See also **Figure 6**

bioRxiv preprint doi: https://doi.org/10.64898/2026.05.01.722283; this version posted May 5, 2026. The copyright holder for this preprint (which was not certified by peer review) is the author/funder, who has granted bioRxiv a license to display the preprint in perpetuity. It is made available under aCC-BY 4.0 International license.

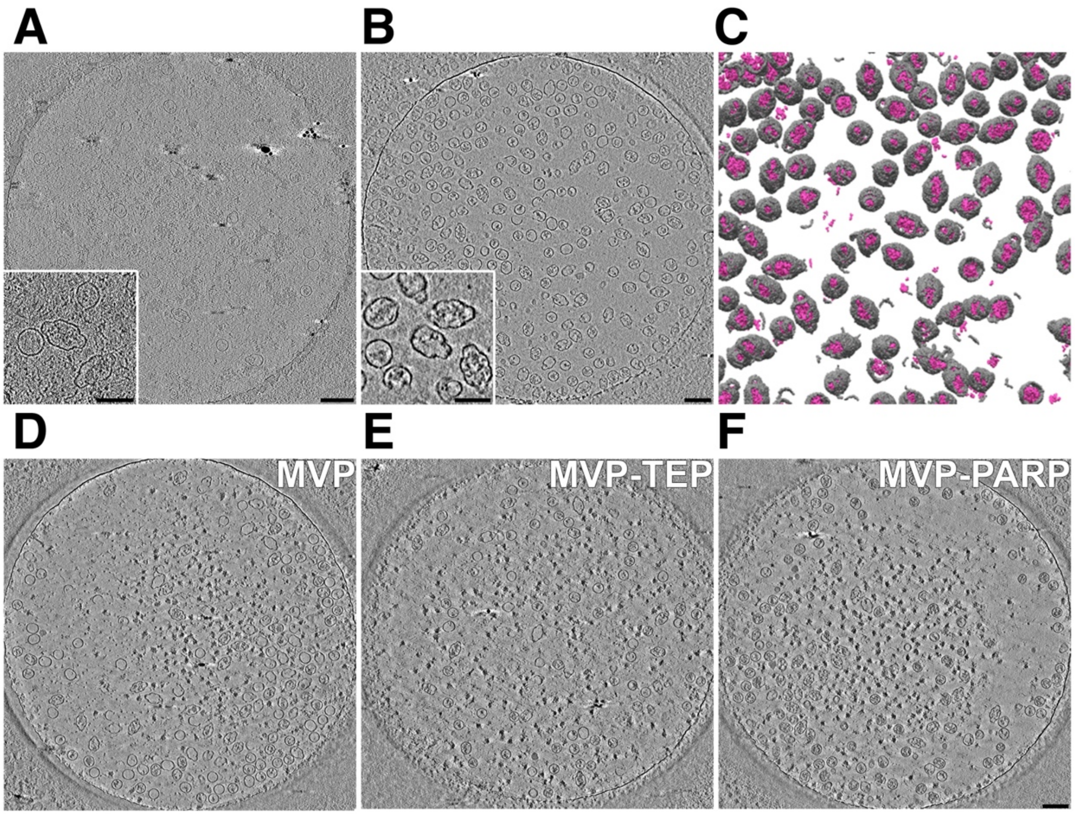

Figure S13. Cryo-ET analysis of reconstituted RNA vaults

(A–B) Central slices from reconstituted vault tomograms acquired using either a conventional dose-symmetric tilt scheme **(A)** or the high-contrast tilt scheme used in this study **(B)**. Insets show magnified views of representative particles from each tomogram. The high-contrast acquisition enhances visualization of lumenal features relative to conventional dose-symmetric collection.

(C) Isosurface renderings of reconstituted vault particles segmented from a high-contrast tomogram. Segmentation was performed in Dragonfly using independently trained models for the vault exterior and interior. Higher-magnification views of representative particles from this dataset are shown in **Figure 6B**.

(D–F) Central slices (5 nm thick) from high-contrast tomograms of reconstituted MVP-only **(D)**, MVP–TEP1 **(E)**, and MVP–PARP4 **(F)** vault particles, illustrating composition-dependent differences in lumenal density under identical imaging conditions. Scale bars represent 10 nm.

See also **Figure 6**
